# Complete Genome Sequence of *Methanohalophilus halophilus* DSM 3094^T^, Isolated from a Cyanobacterial Mat and Bottom Deposits at Hamelin Pool, Shark Bay, Northwestern Australia

**DOI:** 10.1128/genomeA.01604-16

**Published:** 2017-02-16

**Authors:** Stéphane L’Haridon, Erwan Corre, Yue Guan, Manikandan Vinu, Violetta La Cono, Mickail Yakimov, Ulrich Stingl, Laurent Toffin, Mohamed Jebbar

**Affiliations:** a Université de Bretagne Occidentale (UBO), Institut Universitaire Européen de la Mer (IUEM)-UMR 6197, Laboratoire de Microbiologie des Environnements Extrêmes (LM2E), Plouzané, France; b CNRS, IUEM-UMR 6197, Laboratoire de Microbiologie des Environnements Extrêmes (LM2E), Plouzané, France; c Ifremer, UMR 6197, Laboratoire de Microbiologie des Environnements Extrêmes (LM2E), Plouzané, France; d Université Pierre et Marie Curie, CNRS, Station Biologique de Roscoff, Plateforme ABiMS, Roscoff, France; e Red Sea Research Center, King Abdullah University of Science and Technology, Thuwal, Saudi Arabia; f Institute for Coastal Marine Environment CNR, Messina, Italy

## Abstract

The complete genome sequence of *Methanohalophilus halophilus* DSM 3094^T^, a member of the *Methanosarcinaceae* family and the *Methanosarcianales* order, consists of 2,022,959 bp in one contig and contains 2,137 predicted genes. The genome is consistent with a halophilic methylotrophic anaerobic lifestyle, including the methylotrophic and CO_2_-H_2_ methanogensis pathways.

## GENOME ANNOUNCEMENT

*Methanohalophilus halophilus* strain Z-7982 (DSM 3094^T^, OCM 160, NBRC 107633) was isolated from a cyanobacterial mat and bottom deposits at Hamelin Pool, Shark Bay, northwestern Australia. It was first described as *Methanococcus halophilus* (1) before Wilharm and coworkers transfered the strain to the genus *Methanohalophilus* (2). The strictly anaerobic strain Z-7982 is able to produce methane by reducing methylated compounds, and it grows optimally at 30°C (pH 7) with 7% NaCl.

To gain insight into the role of methylotrophic methanogens in marine environments, the complete genome of *M. halophilus* was sequenced. The DNA extracted from strain Z-7982 was sequenced with a 300-bp paired-end library using Illumina MiSeq (Bioscience Core Lab, King Abdullah University of Science and Technology, Thuwal, South Arabia) and a 100-bp paired-end library using Illumina HiSeq (Beckman Coulter, Inc. Genomics, Danvers, MA). The 14,356,400 paired reads of 300 bp were quality trimmed (Q30) and *de novo* assembled into contigs using SPAdes version 3.6.1 (3). The 75 resulting contigs were then scaffolded with SSPACE version 3.0 (4) using the 51,411,253 paired-end reads of 100 bp. Finally, a fully circularized genome was produced, with an average coverage of approximately 4,724×.

The genome of *M. halophilus* consists of a circular chromosome of 2.02 Mb, with a G+C content of 42.39%. A total of 2,137 protein-coding genes (CDSs) were predicted with the MaGe platform (5, 6), as well as 1 copy of the 16S-23S operon, 2 copies of 5S rRNA, 46 tRNAs, and 1 miscellaneous RNA. Additionally, the genome contains one clustered regularly interspaced short palindromic repeat (CRISPR) loci associated with *cas* gene (cas1).

Rapid Annotations using Subsystems Technology (RAST) (7, 8) allowed the discovery of all core methanogenesis enzymes necessary for the conversion of methylated compounds to methane, which is also supported by experimental microbial growth and methane production after the addition of trimethylamine, dimethylamine, monomethylamine, and methanol as sole carbon sources. It was recently suggested that *Methanohalophilus mahii* strain DAL1 (9) has the genetic potential for hydrogenotrophic methanogenesis (conversion of CO_2_ to methane). *M. halophilus* strain Z-7982 also possesses the core enzymes for CO_2_ fixation, acetyl-coenzyme A (acetyl-CoA) decarbonylase, tetrahydromethanopterin *S*-methyltransferase, and methyl-coenzyme M reductase (*mcr*), but experimentally, no growth was observed under CO_2_ in *M. halophilus* strain Z-7982.

### Accession number(s).

This whole genome has been deposited at GenBank under accession no. CP017921.
